# 27 The MacHAND Performance Assessment: Psychometric Testing of the Short English (MPA-S) and French-Canadian (MPA-SF) Forms

**DOI:** 10.1093/jbcr/iraf019.027

**Published:** 2025-04-01

**Authors:** Zoë Edger-Lacoursière, Tara Packham, Alia Sajjad, Bernadette Nedelec, José A Correa

**Affiliations:** Villa Medica Rehabilitation Hospital and McGill University; McMaster University; McGill University; McGill University; McGill University

## Abstract

**Introduction:**

The MacHAND performance assessment (MPA) is one of only 2 measures of dexterity and hand function based on the quality of the movement patterns and time to complete tasks. The MPA is easy to assemble, based on common grasp patterns, composed of cost-effective everyday objects and includes stereognosis tasks. However, it takes 20 mins to administer, which is challenging in a clinical setting. Item redundancy in a pilot study suggested a reduced version could be generated. Furthermore, a valid hand performance outcome measure is needed for the hand burn population.

**Methods:**

The original MPA’s instruction and scoring manual was revised for language consistency, to update pictures, and provide identical 3D printed versions. The MPA-S was developed using a combined statistical and Delphi approach with rehabilitation experts. After finalizing the MPA-S items, an existing dataset MPA was used to assess internal consistency, test-retest and inter-rater reliability for the traumatic hand injury population, as well as agreement with the MPA. The MPA-S was then translated to French-Canadian (MPA-SF) using a forward-backward process. 50 hand burn survivors completed the MPA-SF, along with the Sollerman Hand Function Test and Burn Hand Outcome Tool to establish construct validity. Sessions were video recorded for intra- and inter-rater analysis, with participants re-tested one week later for test-retest analysis.

**Results:**

For the MPA-S, 10 items were retained for dominant hand testing, 8 for non-dominant. The MPA-S showed good internal consistency (Cronbach’s alpha and 95%CI: dominant: 0.85 (0.78, 0.90), non-dominant: 0.88 (0.79, 0.92)), excellent test-retest reliability (r=0.97, 95%CI (0.84, 1), inter-rater reliability (ICC 0.98 (95%CI (0.97, 0.99)) and the mixed effects limits plots show good agreement with the MPA. When tested with the hand burn population, the MPA-SF showed excellent test-retest reliability (r=0.96, 95%CI (0.94,0.98), intra-rater reliability (ICC 0.99 (0.99, 1)) and inter-rater reliability (pre-calibration exercise: (ICC 0.97 (0.96, 0.97) post-calibration: (ICC 0.98 (0.97, 0.99)) for the total scores.

**Conclusions:**

The MPA 2.0 and MPA-S showed adequate psychometric properties for use with traumatic hand injuries. The MPA-SF demonstrated adequate preliminary psychometric properties for the hand burn population. Construct validity evaluation is ongoing.

**Applicability of Research to Practice:**

All forms of this measure are publicly available, providing a cost-effective, standardized measure for hand function.

**Funding for the Study:**

Fondation des pompiers du Québec, Ordre des ergothérapeutes su Québec (OEQ), and REPAR.

